# Early impact of COVID-19 social distancing on social determinants of health and their effects on mental health and quality of life of Korean undergraduate students

**DOI:** 10.3389/fpubh.2023.1197143

**Published:** 2023-07-03

**Authors:** Kyeongeun Lee, Sola Han, Hae Sun Suh

**Affiliations:** ^1^Department of Regulatory Science, Graduate School, Kyung Hee University, Seoul, Republic of Korea; ^2^Institute of Regulatory Innovation Through Science, Kyung Hee University, Seoul, Republic of Korea; ^3^Health Outcomes Division, College of Pharmacy, The University of Texas at Austin, Austin, TX, United States; ^4^College of Pharmacy, Kyung Hee University, Seoul, Republic of Korea

**Keywords:** COVID-19, pandemic, social distancing, undergraduate, quality of life, stress

## Abstract

**Introduction:**

This study aimed to investigate the association between social determinants of health and perception of COVID-19 social distancing/mental health/quality of life during COVID-19 social distancing in Korean undergraduate students using online survey data augmented with natural language processing.

**Methods:**

An online cross-sectional survey including sociodemographic characteristics, students' perceptions of COVID-19 social distancing, and social determinants of health was conducted between July and November in 2020. We conducted logistic regression analysis to investigate the relationship between social determinants of health (independent variables) and perceptions of COVID-19 social distancing, mental health, and quality of life (dependent variables). This association was augmented using sentiment analysis and word clouds by visualizing open-ended comments on COVID-19 social-distancing policies.

**Results:**

Data were collected from 1,276 undergraduate students. Participants who experienced negative impacts on their social-networking activities due to COVID-19 social distancing were at significantly higher odds to perceive COVID-19 social distancing as not being beneficial [odds ratio (OR) = 1.948, 95% confidence interval (CI) 1.254–3.027], to have increased stress levels (OR = 1.619, 95% CI 1.051–2.496), and to experience decreased quality of life over 5 weeks (OR = 2.230, 95% CI 1.448–3.434) against those who answered neutrally. In contrast, Participants who reported positive perceptions of social-networking activities during the COVID-19 pandemic had lower odds of feeling depressed or anxious (OR = 0.498, 95% CI 0.278–0.894) and reporting a low quality of life over 5 weeks (OR = 0.461, 95% CI 0.252–0.842) compared to those who reported neutral perceptions. Furthermore, the results of the word cloud and sentiment analyses showed that most students perceived social distancing negatively.

**Conclusions:**

The government's social-distancing policy to prevent the spread of COVID-19 may have had a negative impact, particularly on undergraduate students' social-networking activities. This highlights the need for greater social support for this population, including access to psychotherapeutic resources, and improvements in policies to prevent infectious diseases while still maintaining social connections.

## 1. Introduction

The World Health Organization (WHO) declared the novel coronavirus disease (COVID-19) a pandemic on March 11, 2020 (1). The WHO strongly recommended quarantine as one of the most effective measures against the contagious outbreak of the disease worldwide (2). Quarantine has been defined by the Centers for Disease Control and Prevention (CDC) as the segregation and restraint of individual movement to prevent people potentially at high risk of exposure to infectious diseases from transmitting the disease (3). Furthermore, the CDC recommended a physical distance of at least six feet to minimize physical interactions between people who are carriers but have not yet been identified or quarantined, which is also interchangeably defined as social distancing (4).

A previous observational study indicated that quarantine, social distancing, and isolation cause anxiety, anger, and depression (5). Brooks et al. reported that quarantine and social distancing psychologically affect people, resulting in stress symptoms, confusion, anger, and fear (2). Researchers have also demonstrated an association between younger age and negative psychological impacts (2). Exposure to negative psychological stress for a long time could be associated with changes in social determinants of health, defined as “conditions in the places where people live, learn, work, and play” by the CDC (6). Social determinants of health are essential to public health outcomes, particularly focusing on mental health (7). This is because various changes in individual conditions of daily life can affect individual psychosocial factors (7).

In the case of South Korea, the Korean government raised the alert level from orange to red on February 23, 2020. This resulted in a ban on gatherings of five or more people, the closure of all schools, and recommendations for telecommuting and social distancing (8). South Korea continued to implement social distancing policies, including restrictions on private gathering sizes, until April 18th, 2022 (9). One study, which used the 2020 Health Survey of Korean adults, found that social determinants of health (e.g., socioeconomic statuses such as age and income, education level, marital status, hypertension, eating habits, and social support) were associated with COVID-19 infection (10). Since Korea implemented strong social distancing policies to prevent the spread of COVID-19, it would be meaningful in the global context to understand the impact of the social determinants of health on undergraduate students' perception of COVID-19 social distancing, mental health, and quality of life.

Korean undergraduate students have undergone significant social and educational changes. They undertook online classes and were unable to meet their professors and classmates in the places where they had learned. Owing to the changes in the Korean government's social-distancing policy, university students experienced confusion due to the inconsistent policies of educational institutions regarding schedules for examinations or school closures. In the context of the pandemic, uncertain and anxious circumstances may have negatively affected the psychological health of undergraduate students (11). In fact, another study examining nurse interns found that the COVID-19 pandemic, which was an unprecedented crisis of such magnitude that has not occurred in a century, caused them to experience stress, trauma, and mental health issues, resulting in a negative impact on their quality of life (12). In addition, a longitudinal study showed that undergraduate students without preexisting mental health problems experienced mental health deterioration during social isolation due to COVID-19 (13). Few studies have analyzed the psychological impact of social distancing and COVID-19 on adults in Korea (14–16).

However, it has rarely been reported among undergraduate students which social determinants of health have been affected by COVID-19 social distancing and their impact on mental health. The present study aimed to evaluate the association between the social determinants of health affected by the COVID-19 social distancing among undergraduate students and their perceptions of COVID-19 social distancing, mental health, and quality of life. We also visualized open-ended comments on COVID-19 social-distancing policies using natural language processing to augment the association.

## 2. Materials and methods

### 2.1. Study design and participants

To assess the perceptions of COVID-19 social distancing among undergraduate students in Korea, a cross-sectional, anonymous online survey was conducted between July and November 2020. The survey was distributed via the online platform (healthbit.com) by the Pusan National University Students' Association, using a convenient non-probability sampling method.

The survey questionnaire was originally developed by Leeza Osipenko as part of a LockDown Project and subsequently piloted with 20 students and staff members from both national and international locations (17). All respondents voluntarily participated in a 15 minutes online survey and were allowed to submit the survey only once. The inclusion criteria for this study were individuals who met the following three conditions: (a) aged 18 years or above, (b) undergraduate students, and (c) willing to participate voluntarily. Exclusion criteria comprised (a) individuals who did not agree to participate (*n* = 6), and (b) those who were not undergraduate students or did not provide their student status (*n* = 288).

We used a sample size calculator to calculate the necessary representative target sample size for achieving the study objectives with sufficient statistical power (18). The calculator determined that a sample size of 601 participants would be needed, considering a margin of error of ±4%, a confidence level of 95%, a response distribution of 50%, and a total population size of 2,633,787 people, which was the total number of university students in Korea in 2020 (19).

This study was approved by the Institutional Review Board of Pusan National University (PNU IRB/2020_62_HR).

### 2.2. Study instruments

The questionnaire consisted of three parts: sociodemographic characteristics, undergraduate students' perception of the COVID-19 social distancing, and social determinants of health impacted by the social-distancing policy during the COVID-19 pandemic. The self-report survey included dichotomous or Likert scale questions. Sociodemographic characteristics including age, residential area type, accommodation type, sex, household income, and comorbidities were recorded. Undergraduate students' perceptions of the COVID-19 social distancing covered questions about their mental health and quality of life. The social determinants of health included (a) social-networking activities, (b) physical exercise, (c) access to health services, and (d) education. Additionally, open-ended questions were asked on undergraduate students' perceptions of the COVID-19 social distancing.

### 2.3. Social-networking activities

Social-networking activities were assessed with a single question: “Due to the COVID-19 social distancing: (a) My social life was impacted, but, overall, I am/was able to cope owing to other support; (b) My social life has been great, and I managed to stay positive; or (c) My social life was negatively impacted.” Assuming that social distancing to avoid the spread of infection may negatively affect university students in terms of social networking, the answers were matched from (a) to (c) on a Likert scale and coded from 0 to 2. For response (b), as the respondent reported that their social life was great and they managed to stay positive, the overall impact was coded as positive perception as 1 = positive perception. For response (c), as the respondent reported a negative impact on their social life, the overall impact was coded as 2 = negative perception. For response (a), as there was no positive or negative direction in the answer, the overall impact was coded as 0 = neutral perception.

### 2.4. Physical exercise

It was assessed whether the physical exercise pattern was changed due to COVID-19 social distancing and how participants feel about this change through the following six items: “Exercise during COVID-19 social distancing: (a) I do not exercise; no change for me; (b) I cannot exercise, but it does not bother me; (c) I started to exercise more; (d) I get sufficient exercise, and I am satisfied; (e) I can exercise, but it is not how I want it; and (f) I cannot exercise, and it decreases my quality of life.” If respondents answered (c) to (e), their response was coded as 1, indicating a positive perception. Option (c) was considered positive because the respondent started to exercise more, which is a positive impact on their exercise routine. Option (d) was also considered positive because the respondent is satisfied with their current exercise routine. Option (e) was also considered positive because even though it is not their preferred way of exercising, they can still exercise. However, if (f) was selected, it was coded as 2, indicating a negative perception. This is because the respondent cannot exercise and it has decreased their quality of life, which is a negative impact on their physical activity. If respondents selected (a) or (b), the score was coded as 0, indicating a neutral perception. For option (a), it was considered neutral because the respondent did not exercise before, so the pandemic did not impact their exercise routine. Option (b) was also considered neutral because the respondent cannot exercise but is not bothered by it.

### 2.5. Access to health services

This dimension was assessed using a single item: “I was effectively able to access health services”: (a) yes or (b) no. If the respondents selected (a), it was graded 1 as a positive perception. If the respondents answered (b), it was graded 2 as a negative perception.

### 2.6. Education

Five questions related to the “Education” dimension were combined to form a single question on whether undergraduate students were affected by the COVID-19 social distancing. The following questions were asked as yes or no: “I was unable to continue my university work partially or fully (e.g., lab shut down or international station required),” “Exams were postponed/canceled,” “Because of the COVID-19 social distancing, I was not able to continue my education in the near term (after life goes back to normal),” “My university did not progress with the exams/assessments and made a relevant arrangement,” “My university was not supportive in offering services, which enabled me to continue my work/education.” It was operationally defined that participants might have been influenced by the COVID-19 social distancing and its related social phenomenon if they answered “yes” to any of the five questions.

### 2.7. Perception of the COVID-19 social distancing, mental health, and quality of life

Four questions were designed to assess participants' perceptions of the COVID-19 social distancing. Two questions were answered using a dichotomous format as follows: “Social distancing is beneficial for me” and “During the pandemic over five weeks, I felt depressed/anxious.” The others examined the participants' level of stress and quality of life during the pandemic over 5 weeks, through the following statements: “During the pandemic over five weeks, my level of stress” (a) decreased or stayed the same or (b) increased; and “During the pandemic over five weeks, my quality of life:” (a) decreased or (b) increased or stayed the same. The response options for the question “Social distancing is beneficial for me” were coded as 0 for “yes” and 1 for “no”. The response options for the question “During the pandemic over 5 weeks, I felt depressed/anxious” were coded as 0 for “no” and 1 for “yes”. For the stress question, a response of (a) was coded as 0, and (b) was coded as 1. For the quality of life question, option (a) was coded as 1, and option (b) was coded as 0. To assess internal consistency, Cronbach's α was used to evaluate the items. A value >0.6 is generally considered acceptable for internal consistency reliability (20).

### 2.8. Statistical analyses

All variables, except for age, were categorical. Descriptive analyses were used to summarize categorical variables as the number of respondents and percentages, and continuous variables as the mean and standard deviation. Chi-square analysis was conducted to examine the relationship between social determinants of health and participants' perception of the COVID-19 social distancing, mental health, and quality of life variables. Missing values were excluded from the analysis for each survey item.

Logistic regression was used to assess the relationship between the impact of COVID-19 social distancing on social determinants of health (social-networking activities, physical exercise, access to health services, and education) and their influence on undergraduate students' perceptions of COVID-19 social distancing, mental health, and quality of life. The responses to survey questions related to the social determinants of health were used as independent variables, while perceptions of COVID-19 social distancing, mental health, and quality of life were used as dependent variables.

To supplement the results of the multivariate logistic regression, open-ended questions were investigated using word cloud and sentiment analysis with text-mining techniques in addition to natural language processing. The word cloud package was used to analyze frequent words from the open-ended questions. Finally, sentiment analysis was implemented to analyze the sentiments of undergraduate students by linking the KNU Korean Sentiment Lexicon (21). The KNU Korean Sentiment Lexicon, created by Kunsan University in Korea, is an emotional dictionary. It comprises positive, neutral, and negative sentiments used to express emotions. The consensus of three evaluators determined the emotion of each word in this dictionary using a 5-point Likert scale, “very negative,” “negative,” “neutral,” “positive,” and “very positive,” ranging from 2 (very positive) to−2 (very negative). All sentimental expressions were classified as positive, negative, or neutral, depending on the sentiment scores.

Statistical analyses, including descriptive and logistic regressions, were performed using SAS version 9.4 (Cary, NC. SAS Institute Inc.) and R version 4.0.2 (R Foundation for Statistical Computing) for word cloud and sentiment analyses. A *P*-value <0.05 was considered statistically significant.

## 3. Results

### 3.1. Sociodemographic characteristics

A total of 1,570 individuals were invited to participate in the survey, of which 1,276 ultimately met our inclusion criteria. Among the 1,276 participants, 572 (44.8%) were women, and 384 (30.1%) were men, with an average age of 22.4 years. Most participants (62.1%) lived in a large city, and the accommodation type was apartments. Regarding family income, 47.2% (*n* = 602) of the respondents belonged to the lower class. Most participants (71.6%) had no underlying diseases. The sociodemographic characteristics of the participants are presented in Table 1.

**Table 1:** Sociodemographic characteristics.

**Attribute**	***n***	**Percentage (%)**
**Demographics [Student at a university (*****n*****)** = **1,276]**		
Age	974	22.40 ± 3.74^a^
**Live in (Residential area)**		
Countryside/sub	21	1.65%
Large city	792	62.07%
Small city/town	210	16.46%
Missing	253	19.83%
**Accommodation**		
Flat	660	51.72%
House	89	6.97%
Rented room	274	21.47%
Missing	253	19.83%
**Sex**		
Female	572	44.83%
Male	384	30.09%
Others	19	1.49%
Missing	301	23.59%
**Household income**		
High income	158	12.38%
Middle income	139	10.89%
Low income	602	47.18%
Prefer not to say	75	5.88%
Missing	302	23.67%
**Comorbidities**		
Yes	60	4.70%
No	914	71.63%
Missing	302	23.67%

^a^Mean ± standard deviation.

### 3.2. Perceived impact of the COVID-19 social distancing on social determinants of health

Table 2 shows the social determinants of health affected by the COVID-19 social distancing. The chi-square analysis revealed significant correlations between social-networking activities, physical exercise, and participants' perception of these variables. Regarding social-networking activities, 29.1% of participants who answered that COVID-19 social distancing was beneficial also reported a negative impact on their social lives. Of the participants who reported an increase level of stress during pandemic over 5 weeks, 30.0% answered that their social lives were negatively impacted, while 6.3% answered that they managed to stay positive. Among those who reported feeling depressed/anxious during the pandemic over 5 weeks in terms of social-networking activities, 26.7% reported a negative impact. In regards to the decreased level of quality of life during the pandemic over 5 weeks, 29.8% reported a negative impact on their social-network activities, while 5.1% felt positive, and the remaining respondents felt neutral. The internal consistency of the perception of COVID-19 social distancing/mental health/quality of life was found to be acceptable with a Cronbach's α coefficient of 0.62.

**Table 2:** Descriptive analysis on questionnaire of the perceived impact of COVID-19 social distancing on social determinants of health.

**Social determinants of health**	**COVID-19 social distancing was not beneficial** ^**a**^			**Feeling depressed/anxious during pandemic over 5 weeks** ^**a**^			**Level of stress during pandemic over 5 weeks** ^**a**^			**Level of quality of life during pandemic over 5 weeks** ^**a**^		
	**Yes [*****n*** **(%)]**	**No [*****n*** **(%)]**	χ ^2^, ***p***	**Yes [*****n*** **(%)]**	**No [*****n*** **(%)]**	χ ^2^, ***p***	**Decreased/ stayed the same [*****n*** **(%)]**	**Increased [*****n*** **(%)]**	χ ^2^, ***p***	**Increased/ stayed the same [*****n*** **(%)]**	**Decreased [*****n*** **(%)]**	χ ^2^**, p**
**Social-networking activities** ^b^												
Neutral	147 (19.8%)	106 (14.3%)	**43.8**, ** < 0.0001**	97 (12.9%)	165 (22.0%)	**57.4**, ** < 0.0001**	147 (19.3%)	115 (15.1%)	**41.7**, ** < 0.0001**	158 (20.2%)	111 (14.2%)	**67.8**, ** < 0.0001**
Positive	96 (12.9%)	54 (7.3%)		32 (4.3%)	109 (14.5%)		90 (11.8%)	48 (6.3%)		108 (13.8%)	40 (5.1%)	
Negative	123 (16.6%)	216 (29.1%)		200 (26.7%)	147 (19.6%)		132 (17.4%)	228 (30.0%)		131 (16.8%)	233 (29.8%)	
**Physical exercise** ^b^												
Neutral	149(20.1%)	148 (20.0%)	**20.1**, ** < 0.0001**	112(14.9%)	189 (25.2%)	**21.7**. ** < 0.0001**	166 (21.8%)	140 (18.4%)	**14.4, 0.0007**	164 (21.0%)	149 (19.1%)	**20.4**, ** < 0.0001**
Positive	182 (24.5%)	149 (20.1%)		143(19.1%)	187 (24.9%)		162 (21.3%)	171 (22.5%)		193 (24.7%)	152 (19.5%)	
Negative	35 (4.7%)	79 (10.7%)		74 (9.9%)	45 (6.0%)		41 (5.4%)	80 (10.5%)		40 (5.1%)	83 (10.6%)	
**Access to health services** ^b^												
Positive	276 (43.1%)	266 (41.6%)	0.53, 0.47	240 (36.8%)	314 (48.2%)	1.1, 0.30	268 (40.8%)	287 (437%)	0.60, 0.44	297 (44.1%)	275 (40.8%)	0.82, 0.37
Negative	46 (7.2%)	52 (8.1%)		48 (7.4%)	50 (7.7%)		45 (6.9%)	8.7% (15.5%)		48 (7.1%)	54 (8.0%)	
**Education** ^c^												
Yes	43 (6.1%)	33 (4.7%)	1.60, 0.21	33 (4.6%)	45 (6.3%)	0.09, 0.77	44 (6.1%)	32 (4.4%)	2.88, 0.09	43 (5.8%)	35 (4.7%)	0.87, 0.35
No	308 (43.6%)	322 (45.6%)		282 (39.3%)	358 (50.0%)		309 (42.6%)	340 (46.9%)		330 (44.4%)	336 (45.2%)	

The bold indicates the significance of the result.

^a^The sample sizes for the survey questions on “COVID-19 social distancing not being beneficial”, “feeling depressed/anxious during the pandemic over 5 weeks”, “level of stress during the pandemic over 5 weeks”, and “level of quality of life during the pandemic over 5 weeks” were 742, 750, 760, and 781, respectively, with missing value percentages of 42, 41, 40, and 39%. Due to the presence of missing data, the summation of values may not always correspond to the sample size across all variables.

^b^“Neutral” indicated participants were not affected by COVID-19 social distancing. “Positive” indicated participants were positively affected by COVID-19 social distancing. “Negative” indicated participants were negatively affected by COVID-19 social distancing.

^c^“Yes” indicated participants were affected by COVID-19 social distancing.

### 3.3. Association between social determinants of health impacted by COVID-19 social distancing and negative perceptions of the COVID-19 social distancing, mental health, and quality of life

Associations between the social determinants of health impacted by COVID-19 social distancing and how these affected undergraduate students' perceptions, mental health, and quality of life were assessed using logistic regression. Table 3 presents the results of these relationships. Participants negatively influenced by social-networking activities during the COVID-19 crisis were significantly associated with the response that the COVID-19 social distancing was not beneficial (OR = 1.948, 95% CI 1.254–3.027) than those who answered neutrally. In contrast, participants who answered positively on social-networking activities were significantly associated with lower odds of feeling depressed or anxious during the COVID-19 pandemic (OR = 0.498, 95% CI 0.278–0.894) compared with those who answered neutrally.

**Table 3:** Association between social determinants of health and negative perceptions of the COVID-19 social distancing, mental health, and quality of life.

		**The response that COVID-19 social distancing was not beneficial**			**Feeling depressed/anxious during pandemic over 5 weeks**			**Increased level of stress during pandemic over 5 weeks**			**Decreased level of quality of life during pandemic over 5 weeks**		
**Social determinants of health**	**Descriptives**	**OR**	**95% CI**	***p*** **-value**	**OR**	**95% CI**	***p*** **-value**	**OR**	**95% CI**	***p*** **-value**	**OR**	**95% CI**	***p*** **-value**
**Social-networking activities, ref. neutral** ^a^													
Positive	20.2%	0.672	0.382–1.183	0.1687	**0.498**	**0.278**–**0.894**	**0.0195**	**0.555**	**0.313**–**0.982**	**0.0433**	**0.461**	**0.252**–**0.842**	**0.0117**
Negative	45.7%	**1.948**	**1.254**–**3.027**	**0.0030**	1.478	0.955–2.286	0.0797	**1.619**	**1.051**–**2.496**	**0.0290**	**2.230**	**1.448**–**3.434**	**0.0003**
**Physical Exercise, ref. neutral** ^a^													
Positive	63.9%	0.640	0.358–1.142	0.1310	1.128	0.637–1.998	0.6802	0.964	0.545–1.706	0.9004	0.837	0.476–1.473	0.5366
Negative	22.0%	1.733	0.872–3.444	0.1167	**2.433**	**1.254**–**4.718**	**0.0085**	1.369	0.706–2.657	0.3524	1.846	0.952–3.581	0.0697
**Access to health services, ref. positive** ^a^													
Negative	15.3%	1.130	0.657–1.945	0.6584	1.141	0.672–1.936	0.6260	1.066	0.635–1.790	0.8084	0.913	0.539–1.546	0.7352
**Education, ref. yes** ^b^													
No	89.2%	0.953	0.476–1.909	0.8916	0.591	0.301–1.158	0.1254	1.037	0.534–2.016	0.9139	1.090	0.541–2.195	0.8090

CI, Confidence Intervals; OR, Odds Ratios.

The bold indicates the significance of the result.

^a^“Neutral” indicated participants were not affected by COVID-19 social distancing. “Positive” indicated participants were positively affected by COVID-19 social distancing. “Negative” indicated participants were negatively affected by COVID-19 social distancing.

^b^“Yes” indicated participants were affected by COVID-19 social distancing.

Participants who responded negatively to physical exercise had significantly higher odds of feeling depressed or anxious during the COVID-19 pandemic over 5 weeks (OR = 2.433, 95% CI 1.254–4.718) against those who answered neutrally.

Regarding the high level of stress during the pandemic over 5 weeks, participants who responded negatively to social-networking activities had higher (OR = 1.619, 95% CI 1.051–2.496) than those who responded neutrally. In contrast, participants who felt positive about social-networking activities had significantly lower odds of reporting high levels of stress during the pandemic over 5 weeks (OR = 0.555, 95% CI 0.313–0.982). This tendency was also observed in low quality of life during the pandemic over 5 weeks. Participants who responded negatively to social-networking activities had a significant association with the response to a low level of quality of life during the pandemic over 5 weeks (OR = 2.230, 95% CI 1.448–3.434) than those who responded neutrally. In contrast, participants with positive perceptions of social-networking activities during the COVID-19 pandemic were significantly correlated with lower odds of reporting a low quality of life over 5 weeks (OR = 0.461, 95% CI 0.252–0.842).

### 3.4. Visualization of social distancing-related discussions

**Figure 1:**
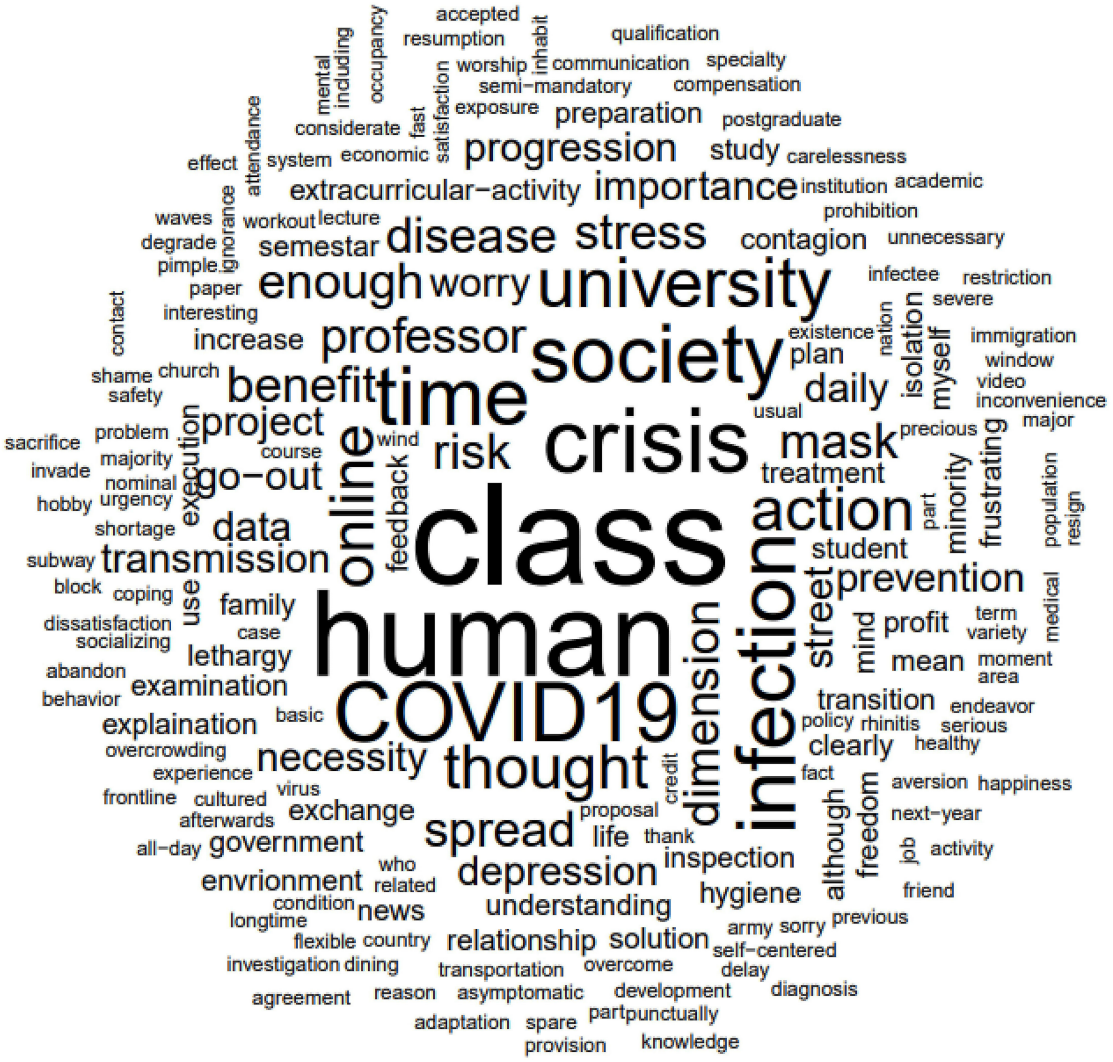
Word clouds showing the most frequently used words across open-ended questions related to COVID-19 social distancing. The word clouds display the words in a central area, where the size of each word is determined by its frequency and importance. Words that are used more frequently and are considered to be more important keywords are shown in larger font sizes, while less frequently used words are displayed in smaller font sizes.

**Figure 2:**
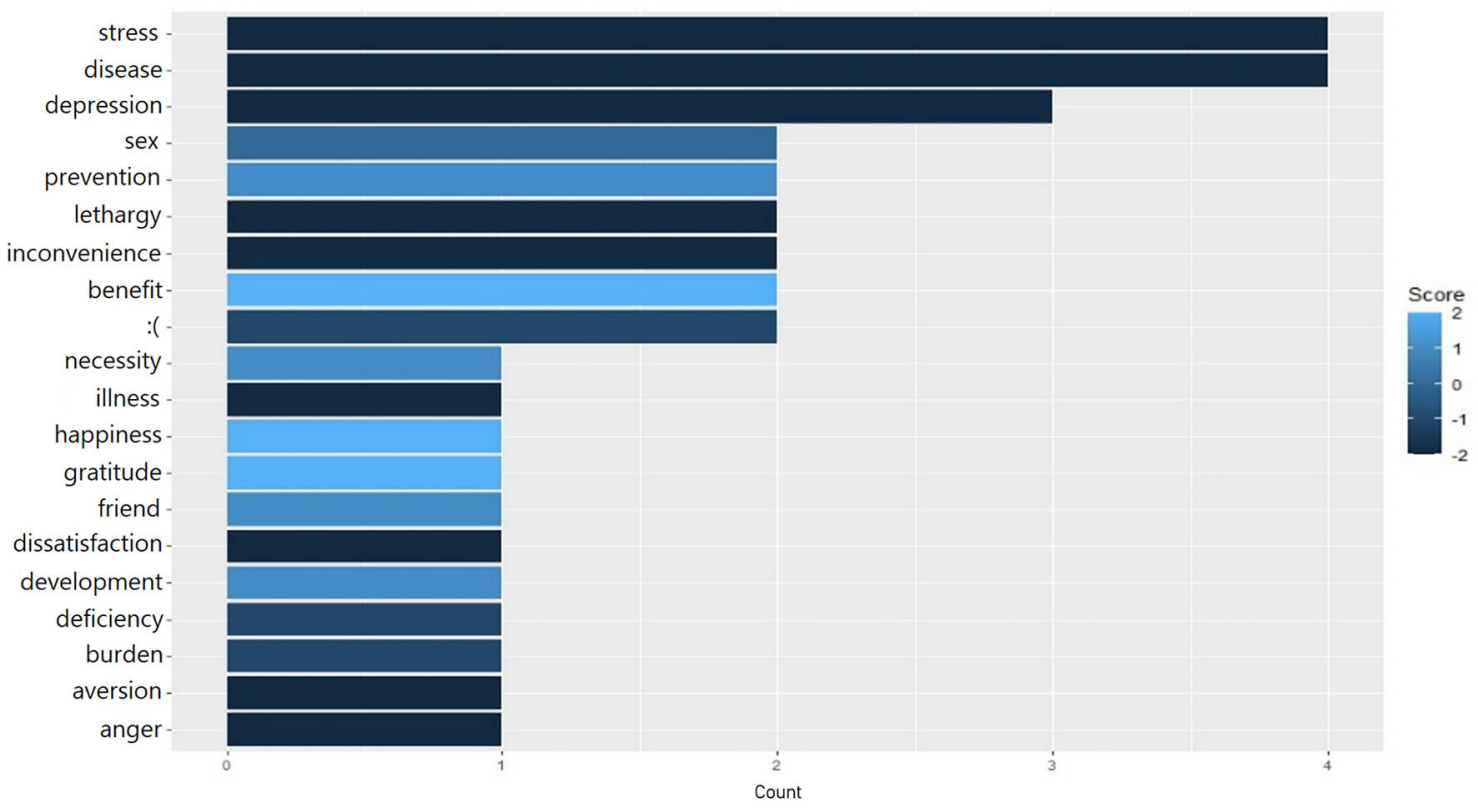
Frequent words across sentiment type toward COVID-19 social-distancing policy among undergraduates. Sentiments were based on the frequency of undergraduates' opinions with respect to the scoring from −2 (very negative) to 2 (very positive).

A total of 212 replies and 743 words remained after removing the background noise and performing lemmatization. Refined words were used to visualize the most frequent and sentiment words for word clouds and sentiment analysis. Figure 1 shows the results of the word cloud visualization based on the open-ended questions related to the COVID-19 social distancing. The highly frequent words are “class,” “human,” and “crisis.” School-related words such as “university,” “online,” and “education” are ranked high, as well as social distance-related words such as “distancing” and “isolation.” Sentiment analysis identified seven words as positive, 12 as negative, and only one as neutral. The most positive sentiment words consisted of “benefit” and “prevention,” whereas “disease,” “stress,” and “depression” occurred in negative sentiment words. The results of the sentiment analysis are shown in Figure 2.

## 4. Discussion

To the best of our knowledge, this study is the first to examine how COVID-19 social distancing affects social determinants of health among Korean undergraduate students, using survey data enhanced by natural language processing. This study contributes to the existing literature in several ways. Most studies have emphasized the psychological impact of online learning on university students (22, 23) or their satisfaction with it (24, 25). The present study found that COVID-19 social-distancing policies had an impact on the social determinants of health among Korean undergraduate students, and this impact was significantly associated with individual perceptions of COVID-19 social distancing, mental health, and quality of life.

In this study, the social determinants of health that were affected by COVID-19 social distancing included social-networking activities, physical exercise, access to health services, and education. Among them, social-networking activities strongly correlated with undergraduate students' perceptions of social distancing during the COVID-19 pandemic. Those negatively influenced by social-networking activities had significant correlations with thoughts that social distancing was not beneficial, increased stress, and resulted in a low quality of life during the pandemic. These results are consistent with the finding of a previous study that longer periods of isolation and inadequate physical space were associated with worse mental health outcomes, including depression (26). In addition, it aligns with the studies that showed younger age groups, in particular, experienced a higher prevalence of loneliness during COVID-19 lockdowns (27, 28). These findings are noteworthy, considering that social isolation as results of social distancing during pandemics might trigger mental health concerns (29, 30), including general psychological distress (31), decreased well-being (32), and lower life satisfaction (32). In contrast, those positively affected by social-networking activities were significantly less likely to feel depressed or anxious, maintain or decrease stress, and increase or maintain their quality of life. It is consistent with the result that Filipino nurses' strong resilience could aid them in dealing with the impact of situational fatigue on their mental health (33). This positive perception can be explained by the following two hypotheses: this may be because greater psychological flexibility and acceptance of difficult thoughts and emotions appear to act as buffers against the negative effects of increased social isolation during the current pandemic (34). In other words, the results support the notion that their high level of resilience can be a valuable asset in mitigating the negative effects of situational fatigue on individual mental health. It may also be explained that a greater amount of time for leisure may allow for better recovery with respect to the university context and the rebuilding of personal resources (13, 35). A recent study reports that people who engage in leisure activities can benefit physical health and well-being and alleviate negative emotions such as sadness, anxiety, and stress (36). Negative changes in physical exercise patterns during the pandemic were also associated with feelings of depression and anxiety. This result is in line with a study that reported a higher likelihood of experiencing anxiety symptoms, depressive symptoms, and high-stress levels among individuals who did not engage in physical exercise during the COVID-19 pandemic (37, 38). It can be explained based on the previous study that physical exercise has various positive effects on the brain, including modulation of neurotransmitter release, enhancement of neurogenesis, anti-neuroinflammatory actions, triggering of neurotrophic factor release, as well as modulation of intracellular signaling to inhibit neuronal dysfunction and promote synaptic plasticity (39). Due to these effects, a negative change in physical exercise patterns may contribute to the development of mental disorders such as depression and anxiety. Based on the open-ended question about individual opinion on COVID-19 social distancing, the frequent words “class,” “human,” and “crisis” are not only shown, but also “distancing” and “isolation” are identified as high ranking. This finding can be linked to the results of the logistic regression in that social isolation as a result of social distancing impacted the perception of undergraduate students about preventing the spread of COVID-19. In addition, regarding COVID-19-related words, “infection,” “mask,” and “spread” were frequently observed. The reason that “mask” was one of the frequent words might be related to the Korean government's policy during the pandemic, which made the public purchase only two masks per week at a uniformly applied price of 1,500 KRW (1.25 USD) in this study period (40).

Sentiments about COVID-19 social distancing had a high proportion of negative responses, suggesting that undergraduate students were unable to face the uncertain and unprecedented public health crises. Negative sentiment words indicated students' negative perceptions of social distancing against COVID-19. The negative emotion-related words such as “stress,” “depression,” and “lethargy” were highly ranked. This result is consistent with this study's finding that undergraduate students are highly likely to feel depressed/anxious, have high levels of stress, and have a low quality of life.

This study had several limitations. First, real-time data were not captured because of its cross-sectional design. Social media data (e.g., Instagram, Twitter, and Facebook) enabled us to analyze public opinion about current topics of interest in real-time; however, it could not provide detailed sociodemographic information and social determinants of health affected by social distancing against COVID-19. Second, the results may not be generalizable to other countries. The Korean government implemented a non-lockdown policy, although governments worldwide have implemented numerous anti-contagion policies to control the COVID-19 pandemic (41). Third, the original survey was developed to quickly investigate a broad range of variables related to the impact of the lockdown on participants. However, the study is limited by the lack of validity tests, such as factor analysis. To address this limitation, we collaborated with at least four native speakers and experts to eliminate any ambiguities in the survey questions and improve its quality. Based on the limitations of this study, future studies could consider using longitudinal designs to capture real-time data and identify changes in perceptions and experiences over time. Additionally, studies could explore the generalizability of findings across different countries and cultures, particularly those with different anti-contagion policies. Future studies could also employ more rigorous validity tests, such as factor analysis, to ensure the quality of survey questions and the accuracy of results. Finally, studies could consider utilizing both social media data and survey data to gain a more comprehensive understanding of public opinion and experiences related to social distancing and COVID-19.

Despite these limitations, the present study has several strengths. A large sample was collected, and an investigation of various dimensions allowed for a detailed analysis. Although previous articles focusing on the psychological impacts of pandemic circumstances have been published (34, 42–44), this study is meaningful because it is the first to observe the influence and importance of social-networking activities in undergraduate students, one of the groups in which social-networking activities are important during the pandemic.

## 5. Conclusions

This study contributes to our collective understanding of the social determinants of health affected by COVID-19 social distancing among undergraduate students, as well as their perceptions of COVID-19 social distancing, mental health, and quality of life.

The impact of COVID-19 social distancing on the social determinants of health can make undergraduates vulnerable to thinking that COVID-19 social-distancing policies are not beneficial to mental health or quality of life. Owing to the government's social-distancing policies to prevent the spread of COVID-19, university students are affected by social determinants of health, such as social-networking activities, resulting in stress, depression, anxiety, and decreased well-being. This highlights the need for greater social support to improve psychotherapeutic settings to help undergraduate students cope with their stress, anxiety, and depression, and to maintain their quality of life. Therefore, it is necessary to evaluate which actions and measures have been taken correctly to prevent infection during this pandemic and what may have a negative impact, to prepare undergraduates to be less psychologically affected in the event of a future pandemic more effectively.

## Data availability statement

Data cannot be shared publicly due to the protection of participants' personal information. Inquiries can be directed to the corresponding author (haesun.suh@khu.ac.k) or Pusan National University Institutional Review Board (irb@pusan.ac.kr).

## Ethics statement

The studies involving human participants were reviewed and approved by Institutional Review Board of Pusan National University. The patients/participants provided their written informed consent to participate in this study.

## Author contributions

KL, SH, and HSS conceptualized this study, interpreted the data, wrote the first draft, and finalized the manuscript. KL and SH performed statistical analyses. HSS collected the primary data and supervised this study. All authors contributed to the article and approved the submitted version.
